# The presence of cerebral and/or systemic endothelial dysfunction in patients with leukoaraiosis - a case control pilot study

**DOI:** 10.1186/s12883-015-0416-z

**Published:** 2015-09-02

**Authors:** Matija Zupan, Mišo Šabović, Marjan Zaletel, Katarina Šurlan Popovič, Bojana Žvan

**Affiliations:** Division of Vascular Neurology, Department of Neurology, University Medical Centre Ljubljana, 2 Zaloška Street, 1000 Ljubljana, Slovenia; Division of Vascular Diseases, Department of Internal Medicine, University Medical Centre Ljubljana, 2 Zaloška Street, 1000 Ljubljana, Slovenia; Clinical Institute of Radiology, University Medical Centre Ljubljana, 2 Zaloška Street, 1000 Ljubljana, Slovenia

## Abstract

**Background:**

In spite of high prevalence and clinical relevance of leukoaraiosis (LA), its pathophysiology is still incompletely understood. Theories of ischaemic genesis and a leaky blood–brain barrier are contradictory yet could share a common denominator–endothelial dysfunction (cerebral, systemic or both), which has not been studied thoroughly in LA.

**Methods:**

Thirty patients with LA (58 years (SD 7)) and 30 gender- and age-matched controls without LA (55 years (SD 6)) were recruited. The vascular risk factors (VRF) were identical in both groups. Cerebral endothelial function was determined by cerebrovascular reactivity to L-arginine (CVR). Systemic endothelial function was determined by flow-mediated dilatation (FMD) of the brachial artery after hyperaemia. All participants underwent a brain MRI to search for radiological signs of LA that was classified according to the Fazekas score. Linear regression was used to explore the correlation between CVR and FMD in patients with LA. A 95 % confidence interval was used. For any statistical test used in the study, *p* ≤ 0.050 was regarded as statistically significant.

**Results:**

We found a marked and significant decrease in both CVR (9.6 % (SD 3.2) vs. 15.8 % (SD 6.1), *p* < 0.001) and FMD (4.8 % (SD 3.1) vs. 7.4 % (SD 3.8), *p* = 0.004) in LA patients compared to controls. Both CVR (7.4 % (SD 3.1) vs. 12.2 % (SD 2.6), *p* = 0.001) and FMD (3.0 % (SD 2.2) vs. 6.4 % (SD 3.1), *p* = 0.011) were significantly decreased in LA subgroup Fazekas 3 compared to subgroup Fazekas 1. CVR and FMD significantly positively correlated (*b* = 0.192, 95 % CI = 0.031–0.354, *p* = 0.02).

**Conclusions:**

The results of our pilot study suggest that patients with LA have a significant impairment of both cerebral and systemic endothelial function that is larger than could be expected based on present VRF. Endothelial dysfunction increases in parallel with LA severity and correlates between cerebral and systemic arterial territory. Overall, our results suggest a so far unknown “intrinsic” generalised endothelial dysfunction in patients with LA that could be involved in LA pathophysiology. This interesting issue needs to be confirmed in larger samples since it could help better understand the mechanisms underlying LA.

## Background

It is well known that leukoaraiosis (LA) is associated with cognitive decline and a higher risk of stroke and death [1]. Given the clinical relevance of LA [2–5], recent years have shown a growing interest in its pathophysiology, which is at present incompletely understood. The concepts of ischaemic genesis [6, 7] and a defective blood–brain barrier [8, 9] seem to oppose each other. Nevertheless, the cerebral endothelial dysfunction might explain both; however, recent studies have not clearly resolved the issue [10, 11]. Thus, in the present studies, the harmful effects of vascular risk factors (VRF) on endothelial function have not been delineated from possible primary endothelial dysfunction in patients with LA. What is more, the relation between cerebral and systemic endothelial function in LA is presently unknown as well.

It has been shown that L-arginine increases cerebral blood flow after intravenous infusion in animal studies as well as in humans [12, 13]. Exogenously derived L-arginine increases the release of nitrous oxide (NO) from the cerebral endothelium, being one of the key vasodilatators. The cerebrovascular reactivity to L-arginine (CVR) is reported to be a reliable marker for cerebral endothelial function [14, 15]. Hence, the method seems to be useful to investigate CVR in patients with LA. Nevertheless, the method has not yet been used to determine cerebral endothelial function in LA. On the other hand, the flow-mediated dilatation (FMD) is a widely used method for the evaluation of systemic endothelial function [16], which has also been applied in LA albeit without concurrently studying cerebral endothelial function [17]. Contrary to previous studies, we aimed at estimating both systemic and cerebral endothelial function in patients with LA.

The aim of this study was to investigate the CVR and FMD in patients with LA. It is well known that several VRF, which are usually found in patients with LA, affect cerebral and systemic endothelial function themselves [18]. In order to determine the role of primary endothelial impairment in LA and to eliminate the confounding effects of VRF on LA, we compared CVR and FMD in LA patients with that of patients with similar VRF without LA and tried to find out whether and how CVR and FMD correlate in patients with LA.

## Methods

The study was performed in two age- and gender-matched groups of patients sharing identical VRF. The inclusion age was between 45 and 65 years. The LA group consisted of patients from neurology outpatient clinic with visible LA in brain magnetic resonance imaging (MRI). All patients in LA group with a history of stroke/minor stroke or transient ischaemic attack (TIA) had the index event at least 6 months before the inclusion. The control group consisted of patients who had undergone brain MRI for other reasons and did not have any radiological signs of LA. The study was approved by the National Medical Ethics Committee of the Republic of Slovenia and all subjects gave written informed consent before inclusion. All the procedures followed were in accordance with institutional guidelines.

The VRF were evaluated based on a standardized interview, encompassing patient history, neurological examination, body mass index determination, laboratory tests and electrocardiography (ECG). The laboratory tests consisted of fasting total cholesterol, high-density lipoprotein (HDL), low-density lipoprotein (LDL), triglycerides (TG) and glucose. Only subjects with normal fasting serum glucose levels (<6.1 mmol/l) based on regular general practice serum glucose measurements, were included. Patients with diabetes were not included in the study. Additionally, laboratory tests included vitamin B_12_, folic acid and thyroid stimulating hormone (TSH) in order to exclude other possible secondary causes of cognitive impairment. Subjects with a known source of cardiogenic embolism based on ECG were excluded from the study.

The colour-coded duplex sonography and power Doppler sonography of the carotid and vertebral arteries were performed in all participants. The intima-media thickness (IMT) was measured bilaterally according to the Mannheim intima-media consensus on the far wall of the common carotid artery [19]. Our final IMT value was based on the mean value of three maximal IMT measurements. Eventual plaques were classified into five standard categories according to their echogenicity. All ultrasonographic measurements were done in a blind fashion by the same experienced ultrasonographer unaware of a particular participant’s status.

A brain MRI without contrast medium was performed in all subjects to look for radiological signs of LA (Philips 1.5T Achieva, coil SENSE-NV-16, Eindhoven, the Netherlands). The scans were systematically evaluated for signs of LA in a double-blind fashion employing two experienced neuroradiologists. The following sequences were used: T2 weighted sequence in the sagittal and transversal plane, echo time (TE) = 100 ms, repetition time (TR) = short, slice thickness 5 mm, matrix 512 × 512; T1 weighted sequence in the transversal plane, TR = 450 ms, TE = 15 ms, slice thickness 5 mm, matrix 320 × 320; FLAIR sequence in the transversal plane, TR = 1100 ms, TE = 140 ms, slice thickness 3 mm, matrix 256 × 256; time of flight (TOF) sequence, TE = out of phase, TR = short, matrix 560 × 560; diffusion imaging, *b* = 0, *b* = 500, *b* = 1000 and an apparent diffusion coefficient (ADC) map. Leukoaraiosis was classified into four subgroups according to the Fazekas score: 0–none, 1–mild punctuate hyperintensive lesions, 2–partly confluent hyperintensive lesions and 3–extensive confluent hyperintensive lesions [20].

The study of endothelial function took place in a quiet room under constant conditions between 8.00 a.m. and 11.00 a.m. after a fasting period of at least 10 h. Flow-mediated dilatation of the right brachial artery was studied according to the recommendations of the International Brachial Artery Reactivity Task Force prior to infusion of L-arginine in order to avoid a possible influence of L-arginine on the determination of FMD [21]. A high-resolution B mode ultrasound system (ALOKA Alpha 10, Tokyo, Japan) with a 10 MHz linear array transducer was used. The patients rested in the supine position for 10 min prior to hemodynamic measurements. The right brachial artery was scanned in the longitudinal section 20 to 100 mm above the antecubital fossa to find the clearest images of the anterior and posterior wall layers. The end-diastolic mean arterial diameter was measured at the end of the diastole period, incident with the R-wave on the simultaneously recorded ECG. At least three cardiac cycles were analyzed for each scan and the measurements averaged. The flow velocity was measured at a fixed incident angle of 68° to the vessel with the range gate of 1.3 mm located in the centre of the artery. The baseline blood flow was estimated by multiplying the velocity time integral of the Doppler flow signal (corrected for the incident angle) by the vessel cross-sectional area. A hyperaemic flow increase was induced by inflation of a blood pressure cuff placed around the forearm to a pressure of 300 mmHg for 4.5 min. The hyperaemic flow was recorded for the first 15 to 20 s and diameter measurements took place 60 to 90 s after cuff deflation. The FMD was expressed as the percentage change in the artery diameter after reactive hyperaemia relative to the baseline scan.

After a 15 min pause, the cerebrovascular reactivity to L-arginine (CVR) was determined. In the preparatory phase, each patient rested for 15 min to achieve a sufficiently relaxed state. The experiment consisted of a 15 min baseline period, a 30 min intravenous infusion of 100 ml of 30 % L-arginine solution and a 15 min interval after the cessation of L-arginine application. The patients were instructed to breathe normally and encouraged not to doze off. The mean arterial velocity (v_m_) was recorded bilaterally in the trunks of both middle cerebral arteries (MCA) through the temporal acoustic windows at a depth of 50 to 54 mm. Two 2 MHz probes were prevented from moving by a mechanical probe holder (DopplerBox, Sipplingen, Germany). Throughout the procedure, the mean arterial blood pressure (MAP) and heart rate (HR) were measured continuously using non-invasive plethysmography (Colin CBM-7000, Komaki-City, Japan). The plethysmograph was connected directly to the Doppler sonograph. The partial pressure of exhaled CO_2_ (CO_2_) was measured by an infrared capnograph (Draeger, Capnodig, Lübeck, Germany), which was connected to a breathing mask and the Doppler sonograph. TCD Multi-Dop X4 (DWL, Sipplingen, Germany) software was used to determine v_m_ during the 10 min rest interval and the 10 min interval following the cessation of L-arginine infusion. The v_m_ was calculated according to the equation:

$$ {\mathrm{v}}_{\mathrm{m}}=\int \mathrm{v}\mathrm{d}\mathrm{t}/\left({\mathrm{t}}_0\hbox{-} {\mathrm{t}}_{10}\right). $$

The CVR was calculated according to the equation:

$$ \mathrm{C}\mathrm{V}\mathrm{R}=\left({\mathrm{v}}_{\mathrm{ms}}\hbox{-} {\mathrm{v}}_{\mathrm{mr}}\right)/{\mathrm{v}}_{\mathrm{mr}}. $$

The v_ms_ denotes the mean arterial velocity after L-arginine infusion and the v_mr_ the mean arterial velocity at rest.

Mean arterial pressure, HR and CO_2_ were calculated for the same intervals as v_m_ using TCD software. All measurements to determine FMD and CVR were carried out by the single experienced investigator who was blind to the patients’ characteristics. The repeatability of TCD measurements of v_m_ upon which CVR is calculated was 95.8 %.

The following variables were statistically analyzed by statistical software SPSS 20.0.0: FMD, baseline diameter of the right brachial artery (diam_b_), posthyperaemic diameter of the right brachial artery (diam_p_), v_m_, MAP, HR, CO_2_ and CVR. The paired *t*-test was used to compare diam_b_ and diam_p_ and additionally v_m_, MAP, HR and CO_2_ before and after intravenous infusion of L-arginine for each group separately. The unpaired *t*-test was used to compare diam_b_, diam_p_, FMD, v_m_, MAP, HR, CO_2_ and CVR-L-arg between patients with LA and those without LA. One-way ANOVA with Bonferroni correction was used to compare FMD, v_m_, MAP, HR, CO_2_ and CVR between the subgroups of LA. Fisher’s exact test was used to examine the significance of the association between categorical variables. Linear regression was used to explore the correlation between CVR and FMD in patients with LA. A 95 % confidence interval (CI) was used. For any statistical test used in the study, *p* ≤ 0.050 was regarded as statistically significant.

## Results

The baseline characteristics of patients in both groups are shown in Table 1. The LA group consisted of 30 patients with LA divided into three subgroups according to the Fazekas score, each containing 10 patients (33.3 %). In only 3 (all Fazekas score 2) out of 30 patients with LA meticulous evaluation for signs of LA yielded slightly asymmetric distribution of LA lesions between both cerebral hemispheres. However, it was not of such amplitude to ascribe a different Fazekas score to both cerebral hemispheres. The control group consisted of 30 patients with similar VRF without LA. The reasons for performing MRI in control patients are outlined in Table 2. The results of laboratory work-up did not show any differences between the LA group and control group (Table 3).

**Table 1:** Baseline characteristics and vascular risk factors in patients with leukoaraiosis (LA) and control group

	LA group (*n* = 30)	Control group (*n* = 30)	*p*-value
Age	58 years (SD 7)	55 years (SD 6)	0.08
Men	17 (56.7 %)	20 (66.7 %)	0.60
Current smoker	5 (16.7 %)	5 (16.7 %)	0.64
Hypertension	19 (63.3 %)	16 (53.3 %)	0.60
Dyslipidemia	13 (43.3 %)	16 (53.3 %)	0.61
Minor stroke/TIA	13 (43.3 %)	0 (0.0 %)	<0.01
Lacunar infarction	9 (30.0 %)	0 (0.0 %)	<0.01
Major ischaemic infarction	1 (3.3 %)	1 (3.3 %)	0.45
Focal neurological deficits	13 (43.3 %)	4 (13.3 %)	0.04
Gait disturbance	12 (40.0 %)	2 (6.7 %)	0.01
Carotid plaques	16 (53.3 %)	10 (33.3 %)	0.19
Ischaemic heart disease	3 (10.0 %)	2 (6.7 %)	0.60
Antihypertensive therapy	19 (63.3 %)	15 (50.0 %)	0.44
Statin therapy	14 (46.7 %)	9 (30.0 %)	0.29
Antiplatelet therapy	20 (66.7 %)	7 (23.3 %)	<0.01
Systolic blood pressure	145.6 mmHg (SD 20.9)	140.1 mmHg (SD 15.0)	0.25
Diastolic blood pressure	88.7 mmHg (SD 10.1)	84.2 mmHg (SD 8.9)	0.07
Body mass index	27.8 kg/m^2^ (SD 4.4)	29.0 kg/m^2^ (SD 5.4)	0.37
Intima-media thickness	0.74 mm (SD 0.11)	0.72 mm (SD 0.11)	0.52

TIA indicates transient ischaemic attack; *SD* standard deviation

**Table 2:** Reasons for performing MRI in control patients

Condition	Number of patients
Focal neurological deficit	4 (13.3 %)
Symptomatic CVD	1 (3.3 %)
Headache	7 (23.3 %)
Extraaxial benign tumour	1 (3.3 %)
ENT reason (vertigo, hearing loss)	17 (56.7 %)

CVD indicates cerebrovascular disease; *ENT* ear, nose, throat

**Table 3:** Results of laboratory work-up in patients with leukoaraiosis and control group

	LA group	Control group	*p*-value
Serum glucose	5.6 mmol/l (SD 1.9)	5.1 mmol/l (SD 0.7)	0.23
Total cholesterol	4.8 mmol/l (SD 1.2)	5.2 mmol/l (SD 0.9)	0.10
HDL	1.2 mmol/l (SD 0.3)	1.3 mmol/l (SD 0.3)	0.19
LDL	2.9 mmol/l (SD 1.0)	3.1 mmol/l (SD 0.9)	0.33
TG	1.5 mmol/l (SD 1.2)	2.2 mmol/l (SD 1.5)	0.08
Vitamin B_12_	256.9 pmol/l (SD 58.8)	291.5 pmol/l (SD 100.1)	0.27
Folic acid	18.1 nmol/l (SD 8.0)	18.1 nmol/l (SD 5.5)	0.99
TSH	1.3 mU/l (SD 0.7)	1.4 mU/l (SD 0.8)	0.73

*HDL* high-density lipoprotein, *LDL* low-density lipoprotein, *SD* standard deviation, *TG* triglycerides, *TSH* thyroid-stimulating hormone

**Fig. 1:**
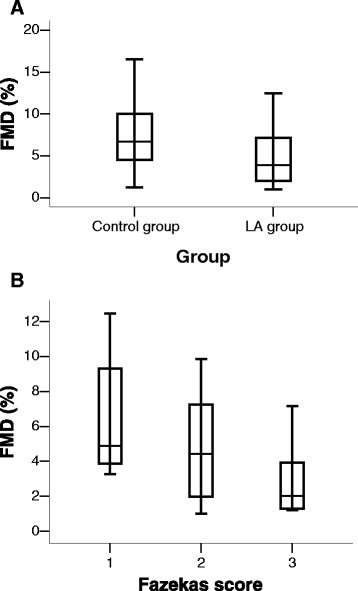
Flow-mediated dilatation (FMD) distribution in control group and leukoaraiosis (LA) group (**a**) and in the subgroups of LA (**b**). SD indicates standard deviation

Within each group, the diam_p_ rose significantly over the diam_b_ in all participants (LA group: diam_b_ = 4.37 mm (standard deviation (SD) 0.94), diam_p_ = 4.58 mm (SD 1.01), *p* < 0.01; control group: diam_b_ = 4.44 mm (SD 0.91), diam_p_ = 4.76 mm (SD 0.94), *p* < 0.01). The FMD was markedly and significantly diminished in patients with LA compared to patients with similar VRF without LA (4.8 % (SD 3.1) vs. 7.4 % (SD 3.8), *p* = 0.004; Fig. 1a). The FMD was markedly and significantly diminished in the subgroup Fazekas 3 compared to the subgroup Fazekas 1 (3.0 % (SD 2.2) vs. 6.4 % (SD 3.1), *p* = 0.01; Fig. 1b).

While the resting v_m_ and that after L-arginine infusion were moderately albeit significantly higher in control group compared to LA group, resting MAP, HR and CO_2_ did not differ between the two groups (Table 4). The v_m_ significantly increased after L-arginine infusion in both LA group (*p* = 0.002) and control group (*p* < 0.001) (Table 4). The MAP after L-arginine infusion (MAP_s_) rose marginally albeit significantly over MAP at rest (MAP_r_) in LA group (*p* = 0.03) and control group (*p* = 0.01) (Table 4). The HR after L-arginine infusion (HR_s_) rose moderately and significantly over HR at rest (HR_r_) in LA group (*p* = 0.002) and control group (*p* < 0.001) (Table 4). The CO_2_ after L-arginine infusion (CO_2s_) did not change significantly compared to CO_2_ at rest (CO_2r_) in LA group (*p* = 0.19) and control group (*p* = 0.40) (Table 4).

**Table 4:** Absolute values of parameters before and after L-arginine infusion in patients with leukoaraiosis (LA) and control group

Parameter	LA group	Control group	*p*-value
v_mr_	53.2 cm/s (SD 14.1)	63.0 cm/s (SD 8.1)	0.002
v_ms_	57.3 cm/s (SD 18.3)	72.9 cm/s (SD 9.5)	<0.001
MAP_r_	103.5 mmHg (SD 19.4)	101.5 mmHg (SD 9.9)	0.63
MAP_s_	106.5 mmHg (SD 20.0)	106.1 mmHg (SD 12.0)	0.93
HR_r_	64/min (SD 13)	62/min (SD 12)	0.63
HR_s_	71/min (SD 12)	70/min (SD 14)	0.85
CO_2r_	19.1 mmHg (SD 2.8)	20.8 mmHg (SD 5.5)	0.13
CO_2s_	18.6 mmHg (SD 3.1)	21.1 mmHg (SD 5.4)	0.05

CO_2r_ indicates partial pressure of carbon dioxide at rest; *CO*_*2s*_ partial pressure of carbon dioxide after L-arginine infusion, *HR*_*r*_ heart rate at rest, *HR*_*s*_ heart rate after L-arginine infusion, *MAP*_*r*_ mean arterial pressure at rest, *MAP*_*s*_ mean arterial pressure after L-arginine infusion, *SD* standard deviation, *v*_*mr*_ mean flow velocity in middle cerebral arteries at rest, *v*_*ms*_ mean flow velocity in middle cerebral arteries after L-arginine infusion

**Fig. 2:**
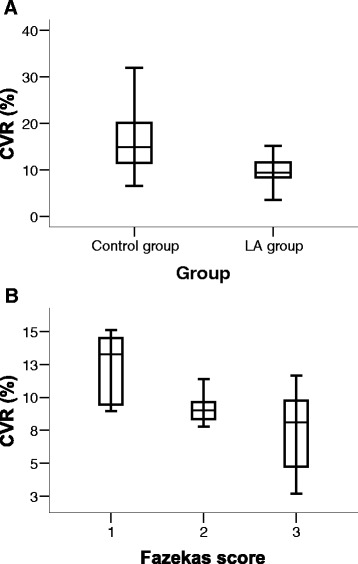
Cerebrovascular reactivity to L-arginine (CVR) distribution in control group and leukoaraiosis (LA) group (**a**) and in the subgroups of LA (**b**). SD indicates standard deviation

**Fig. 3:**
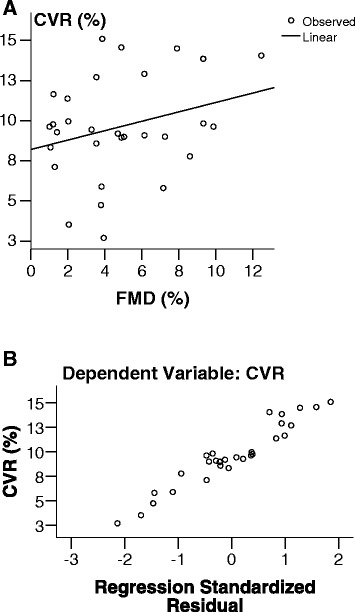
Linear regression showing positive correlation between cerebrovascular reactivity to L-arginine (CVR) and flow-mediated dilatation (FMD) (**a**) and the display of regression standardised residuals (**b**) in patients with leukoaraiosis

The CVR was markedly and significantly lower in patients with LA compared to patients with similar VRF without LA (9.6 % (SD 3.2) vs. 15.8 % (SD 6.1), *p* < 0.001; Fig. 2a). Two out of 3 patients with asymmetric distribution of LA (all belonging to the Fazekas 2 score) expressed modestly lower CVR in the hemisphere with more pronounced LA. However, none of them had significant carotid disease. Patients with Fazekas 3 score had significantly and markedly lower CVR compared to patients with Fazekas 1 score (7.4 % (SD 3.1) vs. 12.2 % (SD 2.6), *p* = 0.001; Fig. 2b) and Fazekas 2 score as well (9.2 % (SD 1.9), *p* = 0.008; Fig. 2b). Linear regression showed a significant positive correlation between CVR and FMD in patients with LA (*b* = 0.192, 95 % CI = 0.031–0.354, *p* = 0.02; Fig. 3). In all patients we observed the increase in both posthyperaemic brachial artery diameter (FMD) and v_m_ after L-arginine infusion (CVR).

## Discussion

The main findings of our study are significantly diminished CVR and FMD in LA patients compared to patients with identical VRF but without LA. Moreover, CVR and FMD correlated positively in patients with LA. Furthermore, within the LA group, CVR was significantly lower in Fazekas 3 patients compared to Fazekas 1 and even Fazekas 2 patients, whereas FMD was significantly lower in Fazekas 3 compared to Fazekas 1 patients. Overall, our results suggest that in patients with LA both cerebral and systemic endothelial functions are impaired to the degrees that are much higher than could be expected based on present VRF. These results seem to reveal a so far unreported, more than expected additional impairment of cerebral endothelial function alongside with systemic endothelial function.

Flow-mediated dilatation had been reported to be diminished in patients with LA in previous studies [13, 22, 23] which is in concordance with our findings. Similarly, Pretnar-Oblak et al. [24, 25] and Kim et al. [26] found additionally impaired FMD in patients with lacunar infarctions (LI) compared to patients with similar VRF without LI. To the best of our knowledge, this is the first published study applying CVR to evaluate cerebral endothelial function and comparing it to FMD in LA. Since endothelial dysfunction of the non-cerebral [15, 27] as well as cerebral arteries [25] in patients with VRF had been already proven, a diminished CVR was expected in both groups. What is intriguing, though, is that our study showed additionally impaired CVR in LA. This may reflect additional or even primary impairment of cerebral endothelial function in LA, regardless of the presence of VRF. However, this is contradictory to the results of a former study of our group, where patients with LI did not show any additional impairment of cerebral endothelial function in comparison to patients with similar VRF but no LI [25]. Although LA and LI share some common characteristics and are both part of a wider spectrum, namely cerebral small vessel disease, one should keep in mind the fact that LA and LI are different entities. The differences in CVR between LA and LI could possibly be explained by the greater extent of rarefaction of brain white matter in the former. The additionally lower CVR in LA compared to LI, regardless of the presence of VRF, could reflect the idea of the primary endothelial dysfunction in LA.

Several authors have studied the CVR in stroke patients, but the results have been contradictory. Some have described lower CVR after stroke in comparison to healthy subjects [14, 25, 28], whereas others have found an increase in CVR [29]. It is known that several mechanisms are involved in the pathogenesis of stroke, so it is possible that patient selection was crucial to the heterogeneity of the results. Micieli et al. included patients with symptomatic internal carotid artery stenosis [28, 30], whereas Žvan et al. included patients with large MCA territory infarctions [14]. Pretnar-Oblak et al. included patients with LI [25] and Zimmermann et al. a rather undefined group of patients with smaller infarctions and transient ischaemic attacks [29]. Interestingly, the CVR was enhanced in patients with cerebral autosomal dominant arteriopathy with subcortical infarctions and leukoencephalopathy (CADASIL) compared to healthy controls [11], which is contradictory to our results. In our study, a homogeneous group of patients with ischaemic LA was included regarding the clear presence of similar VRF, which makes the probability of other aetiologies of LA very unlikely but still possible.

The response to L-arginine in patients with LA is complex. It is well known that the endothelial enzyme nitric oxide synthase (NOS III) uses L-arginine as a precursor for the synthesis of NO [31], which is the main vasodilatator and blood pressure regulator [32]. Vascular risk factors suggest that patients with LA have a reduced synthesis and/or increased oxidative breakdown of NO [27, 33]. It has been shown in animal studies that adaptation to cerebral ischemia leads to NOS III up-regulation [34] and an increase in NO production [35]. Therefore, one expects to find increased response of cerebral arterioles to L-arginine in acute ischemia. However, in LA there is chronic ischemia [36] with rarefaction of brain white matter, sometimes referred to as “incomplete infarction” with putative quantitative endothelial loss, which also means the loss of NOS III. This would explain lower response of cerebral arterioles to L-arginine in LA. On the other hand, studies have been published supporting the role of blood–brain barrier dysfunction in the pathogenesis of LA [8, 9]. The hypotheses of chronic ischemia and a leaky blood brain barrier are not mutually exclusionary since they could be elegantly coupled through cerebral endothelial dysfunction, which could be an initial (primary) event in the pathophysiological cascade leading to LA. This could offer additional basis for further clarifying the putative role of genetic polymorphisms and their neuropathological correlations in LA [37, 38].

The relation between FMD and CVR in patients with LA has not been studied thoroughly thus far. Albeit employing different methodology, cerebral and systemic endothelial dysfunction positively correlated in a study in CADASIL patients [39]. If FMD and CVR truly correlate in LA this may offer possibilities for a simplified estimation of cerebral endothelial function in these patients on the basis of performing solely FMD, which, contrary to complicated and rather inconvenient CVR, is a non-invasive, reasonably easy-to-perform and safe method for the evaluation of endothelial function. From the clinical standpoint, our study may offer a background for further intervention studies aiming at enhancing endothelial function in LA. Whether this reflects in any significant improvement of clinical picture, particularly cognitive domain is a question to be studied in further studies.

It is well known that endothelial dysfunction is a significant pathological condition implicated in the pathophysiology of several vascular disorders. It can never be regarded solely as a surrogate marker of a disease but instead as an “active player” in the pathophysiological mechanisms. We found additionally impaired cerebral and systemic endothelial function in LA patients to a degree not expected on the basis of VRF alone. Not to overlook is our finding that the stage of LA according to the Fazekas score correlates with the degree of endothelial dysfunction. In our view, additional endothelial dysfunction to the degree unexpected solely on the basis of VRF may be implicated in the pathophysiology of LA. There are numerous articles showing or suggesting the presence of impaired endothelial function as a cause or contributor to small vessel disease in general and leukoaraiosis in particular [40–47]. However, in the listed studies neither cerebral nor systemic endothelial functions have been studied together. Also the relations of cerebral and systemic endothelial dysfunction to the severity of LA and the relation between cerebral and systemic endothelial dysfunction still need to be further researched.

One of our study’s limitations is a small number of investigated subjects as a whole, and particularly in view of 10 patients each per Fazekas scores 1, 2 and 3. With a small sample like this we cannot exclude the possibility of false positive results, so they need to be confirmed in larger samples. In light of this, the association between FMD and CVR in our LA patients is weak, but despite such a small number of participants, already significant. The strength of our study lies in the fact that we included a rather homogenous group of relatively young patients with similar VRF, presumably being at initial or moderate stages of LA, which may offer further opportunities for intervention. Nevertheless, it is clear from our data that LA is clinically significant in our patients. The history of stroke, the presence of gait disturbances and focal neurological signs were significantly more frequent in LA group. However, our findings support the hypothesis of an underlying possibly “intrinsic” primary cerebral and systemic endotheliopathy in patients with LA.

## Conclusions

To summarise, our pilot study revealed diminished CVR and FMD correlating positively in patients with LA compared to patients with similar VRF but without LA. This may reflect “intrinsic” generalised endothelial dysfunction regardless of VRF in patients with LA, which could be the initial, primary event in its pathophysiology. However, since this is a preliminary study the results need to be confirmed in larger samples in order to help better understand the mechanisms underlying LA. This may offer streamlined opportunities for future studying of clinical interventions aiming at enhancing endothelial function in patients with LA.

## Abbreviations

ADC: Apparent diffusion coefficient
CADASIL: Cerebral autosomal dominant arteriopathy with subcortical infarctions and leukoencephalopathy
CO_2_: Partial pressure of exhaled carbon dioxide
CI: Confidence interval
CVR: Cerebrovascular reactivity to L-arginine
diam_b_: Baseline diameter of right brachial artery
diam_p_: Posthyperaemic diameter of right brachial artery
ECG: Electrocardiogram
FMD: Flow-mediated dilatation
HDL: High-density lipoprotein
HR: Heart rate
IMT: Intima-media thickness
LA: Leukoaraiosis
LDL: Low-density lipoprotein
LI: Lacunar infarction
MAP: Mean arterial pressure
MCA: Middle cerebral artery
MRI: Magnetic resonance imaging
NO: Nitrous oxide
NOS III: Nitrous oxide synthase
TCD: Transcranial Doppler echosonography
TE: Echo time
TG: Triglycerides
TOF: Time of flight
TR: Repetition time
TSH: Thyroid-stimulating hormone
v_m_: Mean arterial velocity
v_mr_: Mean arterial velocity at rest
v_ms_: Mean arterial velocity after L-arginine infusion
VRF: Vascular risk factors
